# Quantitation of Human Seroresponsiveness to Merkel Cell Polyomavirus

**DOI:** 10.1371/journal.ppat.1000578

**Published:** 2009-09-11

**Authors:** Diana V. Pastrana, Yanis L. Tolstov, Jürgen C. Becker, Patrick S. Moore, Yuan Chang, Christopher B. Buck

**Affiliations:** 1 Laboratory of Cellular Oncology, National Cancer Institute, Bethesda, Maryland, United States of America; 2 Molecular Virology Program, University of Pittsburgh Cancer Institute, University of Pittsburgh, Pittsburgh, Pennsylvania, United States of America; 3 Department of Dermatology, Venerology and Allergy, University Clinic of Würzburg, Würzburg, Germany; University of Colorado, United States of America

## Abstract

Merkel cell carcinoma (MCC) is a relatively uncommon but highly lethal form of skin cancer. A majority of MCC tumors carry DNA sequences derived from a newly identified virus called Merkel cell polyomavirus (MCV or MCPyV), a candidate etiologic agent underlying the development of MCC. To further investigate the role of MCV infection in the development of MCC, we developed a reporter vector-based neutralization assay to quantitate MCV-specific serum antibody responses in human subjects. Our results showed that 21 MCC patients whose tumors harbored MCV DNA all displayed vigorous MCV-specific antibody responses. Although 88% (42/48) of adult subjects without MCC were MCV seropositive, the geometric mean titer of the control group was 59-fold lower than the MCC patient group (p<0.0001). Only 4% (2/48) of control subjects displayed neutralizing titers greater than the mean titer of the MCV-positive MCC patient population. MCC tumors were found not to express detectable amounts of MCV VP1 capsid protein, suggesting that the strong humoral responses observed in MCC patients were primed by an unusually immunogenic MCV infection, and not by viral antigen expressed by the MCC tumor itself. The occurrence of highly immunogenic MCV infection in MCC patients is unlikely to reflect a failure to control polyomavirus infections in general, as seroreactivity to BK polyomavirus was similar among MCC patients and control subjects. The results support the concept that MCV infection is a causative factor in the development of most cases of MCC. Although MCC tumorigenesis can evidently proceed in the face of effective MCV-specific antibody responses, a small pilot animal immunization study revealed that a candidate vaccine based on MCV virus-like particles (VLPs) elicits antibody responses that robustly neutralize MCV reporter vectors in vitro. This suggests that a VLP-based vaccine could be effective for preventing the initial establishment of MCV infection.

## Author Summary

For more than 50 years it has been known that some polyomavirus types can induce cancer in experimental animals. However, associations between the various polyomaviruses known to chronically infect most humans and the development of cancer have been difficult to uncover. Last year, DNA from a new human polyomavirus, called Merkel cell polyomavirus (MCV), was found embedded in an uncommon form of skin cancer called Merkel cell carcinoma. Emerging evidence indicates that most adults display detectable immune responses to MCV, suggesting that most individuals eventually become infected with the virus. In this study, we investigate antibodies that directly bind the protein coat of MCV, thereby obstructing its ability to penetrate cultured cells. We found that the magnitude of antibody responses against MCV varies dramatically among normal adults. Interestingly, patients suffering from MCV-associated Merkel cell carcinoma display uniformly strong antibody responses against the virus. This suggests that the development of Merkel cell carcinoma is preceded by an unusually robust MCV infection. It is currently unclear whether MCV infection may also be associated with additional diseases aside from Merkel cell carcinoma. Quantitation of immune responsiveness to the virus, using techniques reported here, could help identify such links.

## Introduction

The *Polyomaviridae* are a diverse family of non-enveloped DNA viruses named for some family members' ability to cause various types of tumors in experimentally challenged animals. Although BK and JC polyomaviruses (BKV and JCV) are highly prevalent in human populations, neither virus has been clearly shown to cause cancer in humans (reviewed in [1]). A previously unidentified polyomavirus was recently found associated with Merkel cell carcinoma (MCC), a relatively unusual form of skin cancer that tends to strike elderly or immunocompromised individuals ([2], reviewed in [3],[4]). Sequences from this new virus, called Merkel cell polyomavirus (MCV or MCPyV), have been confirmed to be present in a majority of MCC tumors [5]–[8]. The viral DNA is maintained as a circular episome during productive infection but is clonally integrated into the cellular DNA of MCV-positive MCC tumors. Integrated viral genomes carry a characteristic pattern of mutations of the large T antigen gene that produce truncating deletions of the T antigen protein [9]. The mutations abrogate the protein's ability to drive replication of the viral DNA but preserve regions with predicted oncogenic potential. In some integrated viral genomes, deletions also occur in the late region of the virus encoding the viral capsid proteins [5],[10]. Taken together, the available evidence suggests that nonproductive integration of MCV genomic DNA into the host cell's DNA is an etiologic factor underlying the development of most cases of MCC.

Recent serological studies using recombinant MCV capsid proteins have shown that about 50–80% of adults display detectable MCV-specific antibody responses [11],[12]. This suggests that MCV infection is common, but only rarely leads to MCC. Although a majority of adults are seropositive for MCV, our initial serological studies suggest that some individuals display stronger humoral responses to MCV than others. To more accurately quantitate MCV-specific serum antibody responses in human subjects, we developed an assay for measuring antibody-mediated neutralization of cellular transduction with an MCV-based reporter vector. The assay employs very low viral particle doses, allowing improved accuracy and reproducibility compared to previously-reported MCV serological methods.

Unlike enzyme-linked immunosorbent assays (EIAs), which simultaneously measure both neutralizing and non-neutralizing antibodies, viral neutralization assays have the useful feature of measuring only the subset of antibodies that are likely to confer protection against infection. Neutralization assays have therefore been used for characterizing candidate vaccines [13]. Although VLP-based vaccines against viruses such as human papillomavirus (HPV) and hepatitis B virus are highly immunogenic, it appears that VLPs based on some polyomavirus types can be poorly immunogenic in animal model systems [14]. Using the MCV reporter vector-based neutralization assay, we show that MCV VLPs elicit robust functional antibody responses and thus could potentially be employed in vaccines aimed at preventing MCV infection.

## Results

### Development of MCV-based reporter vectors

Isolation of infectious MCV virions has not yet been reported. To simulate MCV infection in vitro, we generated gene delivery vectors employing the VP1 and VP2 capsid proteins of MCV. The MCV reporter vectors were produced by transfecting human embryonic kidney-derived 293TT cells [15] with expression plasmids carrying codon-modified versions of MCV VP1 and VP2 genes of MCV isolate 339 [2],[12]. For initial optimization experiments, the VP1 and VP2 expression plasmids were co-transfected with a reporter plasmid encoding GFP. The transfected cells produced high yields of capsids with a VP1∶VP2 ratio of about 6∶1 [12]. A fraction of the particles encapsidated the GFP reporter plasmid. The GFP transducing potential of the MCV-based reporter vector particles was titered on HeLa cells, which were found to be permissive for transduction with the GFP reporter gene.

Previously-identified polyomaviruses encode a minor capsid protein, VP3, whose translation initiates from an in-frame methionine (Met) codon within the VP2 open reading frame. However, MCV lacks the conserved Met-Ala-Leu motif that forms the amino-terminus of all previously described polyomavirus VP3 proteins. We generated expression plasmids encoding possible alternative VP3 proteins initiated from MCV VP2 Met_46_ or Met_129_ codons. While inclusion of VP2 improved the infectivity of the MCV reporter vector by about five-fold, compared with using VP1 alone, inclusion of the candidate VP3 expression constructs either slightly reduced or did not affect reporter vector infectivity (data not shown). The results suggest that, in contrast to other polyomaviruses, MCV may not encode a functional VP3 protein.

It has recently been shown that bacterially-expressed VP1 capsomers based on MCV isolate 350 are serologically distinct from MCV339 capsomers [11]. Like MCV339, MCV350 was isolated from an MCC tumor. We attempted to generate reporter vectors based on the MCV350 VP1 protein. However, the VP1 protein of MCV350 was rapidly degraded to undetectable levels in 293TT cell lysates (Figure S1). Attempts to purify MCV350 capsids by ultracentrifugation were similarly unsuccessful (data not shown). The results indicate that MCV350 encodes a structurally defective VP1 protein, possibly due to mutations arising during tumorigenesis. This concept is consistent with the fact that MCV350 VP1 residues His_288_, Ile_316_ and Asn_366_ differ from the consensus Asp, Arg or Asp residues (respectively) that are highly or absolutely conserved among all known polyomaviruses, including MCV339 and a variety of more recently described MCV VP1 isolates [16].

### Development of an MCV neutralization assay

The transducing potential of a viral vector can typically be blocked by antibodies capable of neutralizing the virus on which the vector is based. To develop a reporter vector-based MCV neutralization assay, we employed a highly sensitive *Gaussia* luciferase (Gluc) reporter gene. 293TT cells [15], which stably express SV40 large T antigen, were used as an infection target. Successful transduction of 293TT cells results in T antigen-mediated amplification of the transduced Gluc reporter plasmid, which carries the SV40 origin of replication. The MCV-Gluc/293TT assay is highly sensitive, with MCV-Gluc reporter vector doses of 80 pg of VP1 per well (roughly 8 pM with respect to VP1 or roughly 100 virions per cell) yielding signal to noise ratios of 1000∶1.

**Figure 1:**
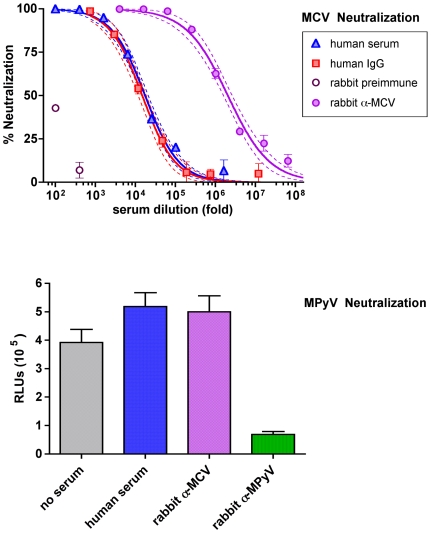
Quantitation of MCV-specific neutralizing antibodies in human serum. Top panel: Pooled human serum and IgG purified out of the pooled serum were serially diluted and tested in an MCV reporter vector-based neutralization assay. Rabbit sera collected before or after immunization with MCV VLPs were also tested. A sigmoidal dose-response curve was fitted to the data points. Dashed lines show 95% confidence intervals. Bottom panel: serum samples indicated were diluted 1∶500 and tested for inhibition of MPyV-mediated Gluc transduction (measured in relative light units, RLUs). Error bars in both panels show standard error of the mean.

A pooled human serum sample was serially diluted and tested for the ability to neutralize MCV vector-mediated transduction of the Gluc gene into cells. 50% neutralizing titer (EC_50_) was calculated by fitting a sigmoidal dose-response curve to luminometric values for the dilution series. The calculated EC_50_ for the pooled serum occurred at a 17,900±2500-fold serum dilution (Figure 1). Serum from a rabbit inoculated with MCV VLPs (see below) also robustly neutralized the infectivity of the MCV-Gluc reporter vector, while preimmune serum from the rabbit was less than 50% neutralizing at the 1∶100 serum dilution. Since the pre-immune rabbit serum showed non-specific neutralizing effects at dilutions less than 1∶100, this dilution was chosen as a cutoff for subsequent work.

A control experiment using IgG purified out of the pooled human serum gave a neutralization curve that overlapped that of the original serum (EC_50_ = 14,900±2200). Conversely, stripping the pooled serum of immunoglobulins reduced the EC_50_ by nearly 40-fold (data not shown). The results demonstrate that the MCV vector-neutralizing activity of serum diluted 1∶100 or greater is entirely or almost entirely attributable to antibodies.

Serological cross-reactivity between BKV and SV40, which occupy a phylogenetic cluster that also includes JCV, has previously been documented (reviewed in [1] ). MCV is part of a different phylogenetic cluster that includes African green monkey B-lymphotropic polyomavirus (LPV) and murine polyomavirus (MPyV). It has long been suspected that an LPV-like virus infects humans [17]. Kean and colleagues have recently confirmed that 10–20% of human subjects display LPV-specific antibody responses in a capsomer-based EIA. The report further demonstrated that antibodies specific for MCV do not cross-react with LPV [11]. To verify that the vector-based MCV neutralization assay is specific for MCV, we developed a neutralization assay based on MPyV, which, in contrast to LPV, is not thought to infect humans. Neither the pooled human serum nor the MCV-specific rabbit serum inhibited transduction of 293TT cells by the MPyV reporter vector (Figure 1). In contrast, the MPyV reporter vector was neutralized by control serum from a rabbit immunized with MPyV VP1 [18]. Similar results were observed when the MPyV reporter vector and sera were applied to murine NIH-3T3 cells (data not shown). We also developed an LPV reporter vector and confirmed the observations of Kean and colleagues that 10% of serum samples from paid donors had very low neutralizing titers to LPV reporter vectors (data not shown). The majority of donors with neutralizing LPV titers did not have significant MCV neutralizing titers, although other sera did (see below). The results demonstrate that neutralizing antibodies in human sera are specific for MCV and not one of MCV's known near relatives.

### Quantitation of MCV-neutralizing antibodies in individual human sera

Under ideal circumstances, the EC_50_ values observed in neutralization assays and VLP-based EIAs reflect the affinity of relevant antibodies for the viral capsid. This requires that the assay conditions satisfy the assumptions of the law of mass action. This concept was first put forward in 1933 by Andrewes and Elford as the “percentage law,” which states that the virus-neutralizing titer of an antibody preparation is not affected by the amount of virus, so long as the antibody is in excess over the virus [19]–[21]. In other words, if the concentration of antigen in the assay approaches or is in excess of the affinity constants of the antibody/antigen interactions being measured, antibody is stripped from solution before affinity-driven equilibrium between bound and unbound antibody can be reached. As a consequence, the EC_50_ begins to reflect the dose of antigen, rather than the affinity of the interaction. A straightforward strategy for testing whether a seroassay complies with the percentage law is to examine EC_50_ values for various antigen doses [22]–[24]. Under compliant conditions, the EC_50_ is insensitive to antigen dose.

**Figure 2:**
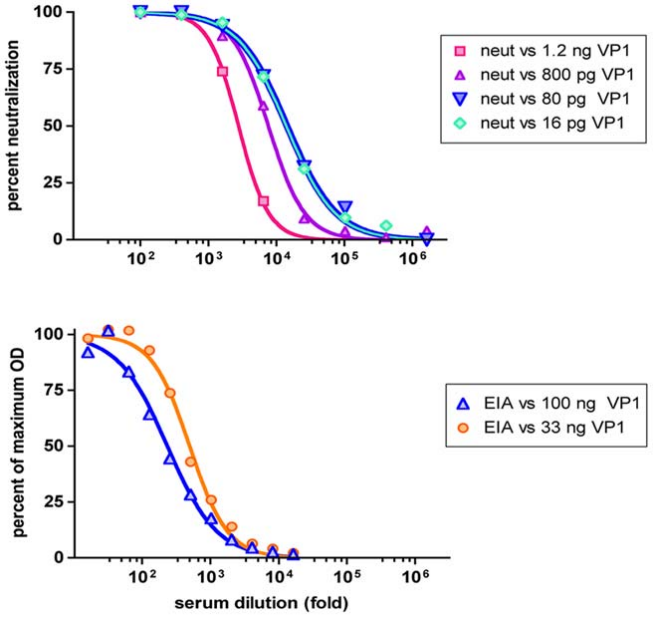
MCV neutralization assay complies with the percentage law. Pooled human serum was tested in MCV neutralization assays (top panel) or EIAs (bottom panel) employing various doses of VP1 antigen (VP1 mass per assay well indicated in legend). Percent neutralization was calculated by standardization to calculated maximum RLU values. EIA curves were standardized to calculated maximum OD values. In both panels, standard VP1 doses are plotted as dark blue triangles.

Neutralization assays of the pooled human serum using MCV-Gluc doses ranging from 16 to 240 pg of VP1 per well gave neutralization curves that were not significantly different, with EC_50_ values ranging from 15,600 to 17,900 (Figure 2). In contrast, the use of MCV-Gluc doses of 800 pg or 1.2 ng of VP1 per well resulted in lower EC_50_ values (7600 and 2800, respectively). VLP-based EIAs using VP1 doses of 100 or 33 ng per well gave dramatically lower EC_50_ values (230 and 460, respectively, Figure 2). The results indicate that, using standard antigen doses, the neutralization assay complies with the percentage law and the EIA does not. Optimized polyomavirus VLP EIA methods use VP1 doses ranging from 6 to 200 ng per well [25]–[27], suggesting that polyomavirus VLP EIA could not be adapted to the <240 pg/well doses required to comply with the percentage law. The data indicate that the neutralization assay offers a more accurate and sensitive measurement of serological responsiveness to MCV than the EIA. The fact that the neutralization assay is insensitive to virion dose would also be expected to make it more reproducible than the EIA.

**Figure 3:**
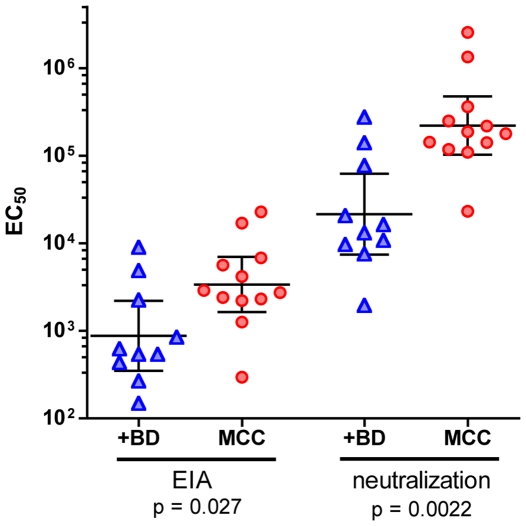
Comparison of EIA to neutralization assay for individual samples. Serum samples from a selected set of 10 robustly seropositive blood donors (+BD) and 12 MCV^+^ MCC patients (MCC) were serially diluted and tested in MCV VLP-based EIA or MCV neutralization assay. EC_50_ values (y-axis) were calculated for each serum sample. Bars within the plots show the geometric mean, with 95% confidence interval.

To further explore the relative accuracy of the neutralization assay, we tested serial dilutions of sera from a selected set of 10 blood donors whose EIA reactivity was robust enough to allow calculation of an EC_50_ value [12]. The blood donors were compared to 12 MCC patients whose tumors were found to harbor MCV DNA sequences. As seen in Figure 3, the neutralization assay allowed improved discrimination between the two groups' seroresponsiveness to MCV. While the EIA suggested a 4-fold difference between the geometric mean titers (GMT) of the blood donor and MCC patient groups (GMT of 876 and 3,390, respectively), the neutralization assay revealed a >10-fold difference (GMT of 21,500 and 222,000, respectively) between the two groups, with correspondingly stronger p values (Figure 3). Furthermore, EIA EC_50_ values for individual subjects were an average of 50-fold lower than their neutralizing EC_50_ values (Figure S2), confirming the greater accuracy of the neutralization assay.

### MCC patients display unusually strong MCV-neutralizing responses

It was striking that subjects with MCC displayed significantly higher neutralizing titers than a selected group of strongly seropositive blood donors (Figure 3). To better characterize this apparent difference; we tested a set of 48 sera from older adults (age range 47–75 years) without diagnosed MCC. The control subject sera were compared to a total of 21 MCV-positive MCC patients (age range 14–95 years). As seen in Figure 3, MCV^+^ MCC patients invariably displayed high titer MCV-neutralizing responses, with a GMT of 160,000. Control subjects, in contrast, showed a broad, continuous distribution of neutralizing titers, with a significantly lower GMT of 2700 (p<0.0001). Only 7/48 (15%) of control subjects displayed titers within or above the interquartile range of the MCV^+^ MCC patient population.

The prevalence of MCV-neutralizing activity in the control subject population was high, with 88% (42/48) of the subjects displaying EC_50_ values falling within the tested range of serum dilutions. It is not clear whether sera with titers below 100 are weakly MCV seropositive or rather contain non-specific neutralizing activity, as was observed for the pre-immune rabbit serum (Figure 1). The neutralization assay results confirm recent findings showing that MCV-specific seroprevalence is common among older adults and suggests that the 67% EIA-based seroprevalence observed in this same group of subjects [12] may have been a slight underestimate.

The presence of high MCV-specific titers in all the MCV-positive MCC patients could, in theory, reflect an immunocompromised state in which latent polyomavirus infections are allowed to resurface, triggering strong virus-specific antibody responses. To test this hypothesis we evaluated sera from the same set of control subjects and MCC patients for the presence of anti-BKV antibodies using a BKV-based reporter vector [28]. There was no apparent correlation between BKV and MCV titers in individual subjects (data not shown), suggesting a lack of general reactivation of polyomaviruses as well as a lack of cross-reactivity between the two virus types in the neutralization assays. The BKV GMT was 5,100 for control subjects and 2,300 for MCV-positive MCC patients (Figure 3). This slight difference in titer was not statistically significant.

Sera from a set of six MCC patients whose tumors did not contain detectable amounts of MCV DNA were also tested in the neutralization assay. 4/6 of the MCV^−^ MCC patients displayed very low titers in the neutralization assay (Figure S3).

**Figure 4:**
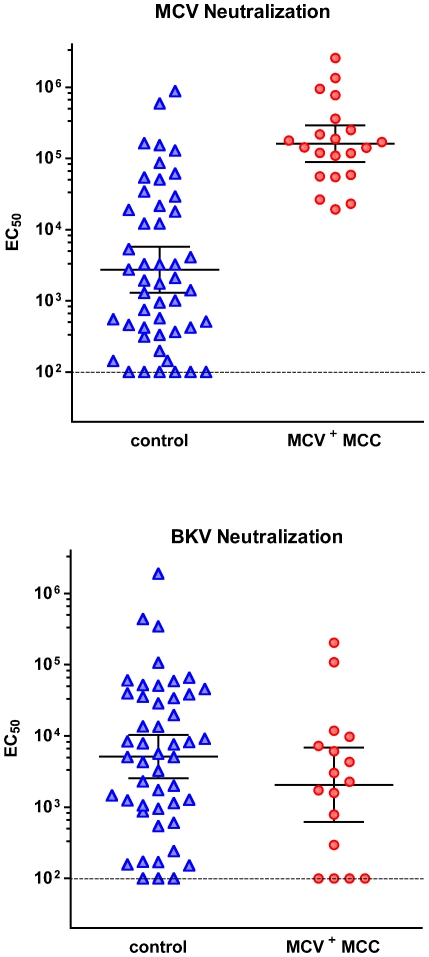
MCV-positive MCC patients invariably display strong MCV-neutralizing antibody responses. Sera from a set of subjects without MCC (control) and MCV^+^ MCC patients were serially diluted and tested using the neutralization assay. EC_50_ values for each serum are shown (y-axis). Black bars show the geometric mean EC_50_ (with 95% confidence interval) for each group. Samples with projected EC_50_ values falling below the bottom of the assay's standard dilution series (dotted line) were plotted at 100. Top panel: Anti-MCV titers for 48 controls and 21 MCV^+^ MCC patients. The difference between the two groups was statistically significant (p<0.0001, Mann-Whitney non-parametric 2-tailed t test). Bottom panel: Anti-BKV titers for 48 controls and 16 MCV^+^ MCC patients. The difference between these two groups was not statistically significant.

The incidence of MCV seroresponsiveness has been shown to increase with subject age, reaching an apparent maximum prevalence in late adulthood [11],[12]. Age-specific trends in the MCV-neutralizing titers of the control subjects shown in Figure 4 were not evident, perhaps in part because the distribution of ages is clustered about the mean (56±5.7 years, Figure S3). Interestingly, adult MCV^+^ MCC patients displayed a marginally significant inverse correlation between subject age and MCV-neutralizing titer (p = 0.0497, Spearman r = −0.4443, Figure S3). The trend is reminiscent of the gradual age-related decline in BKV-specific antibody responses observed in cross-sectional studies of adults [29]. The data indicate that the higher MCV-specific titers of the MCC patients are unlikely to be attributable simply to their more advanced average age relative to the control subjects.

### Investigation of capsid protein expression in MCC tumors

One possible explanation for the higher MCV-specific antibody titers of MCV^+^ MCC subjects could be that the MCC tumor itself serves as a source of MCV capsid protein immunogen. One previous report has documented an MCC tumor that carries a VP1 gene with a large internal deletion that would presumably render the protein incapable of forming intact capsids [5]. The current study suggests that the MCC350 tumor would likewise be genetically incapable of expressing conformationally intact capsids or of making stable protein. However, it remains conceivable that other MCC tumors might produce MCV capsid protein. To address this question, we performed immunohistochemical staining of MCC tumor sections. Since sections of the tumors from subjects on whom the serological studies were performed were unavailable, we selected 10 MCC tumors that had previously scored positive for expression of MCV T antigen [10]. Unfortunately, matched sera for this set of tumors were unavailable.

**Figure 5:**
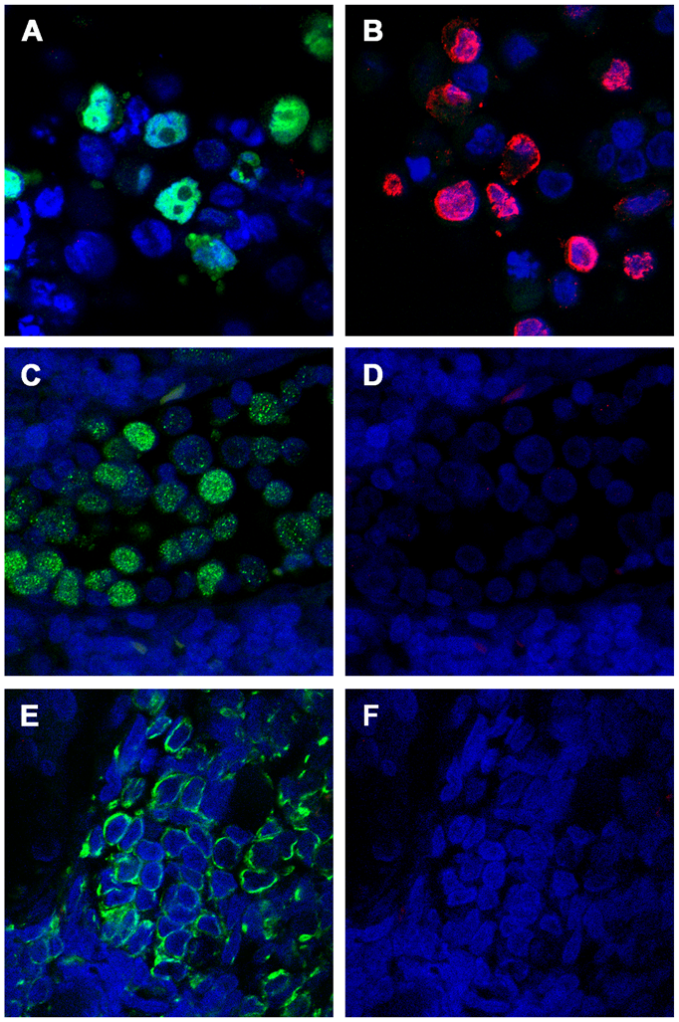
MCC tumors do not express detectable amounts of MCV VP1 protein. Immunohistochemical staining was performed on sections of paraffin-embedded MCC tumors (panels C–F) or paraffin-embedded HeLa control cells transfected with expression constructs encoding MCV T antigen (panel A) or MCV VP1+VP2 (panel B). Cells in panel A were stained with CM2B4, a monoclonal antibody specific for MCV T antigen (green secondary). Cells in panel B were stained with an MCV VLP-specific rabbit serum (red secondary). The MCC tumor section in panels C and D was co-stained with CM2B4 and the rabbit anti-VLP serum. Panel C shows MCV T antigen signal (green secondary), panel D shows MCV VLP signal (red secondary). Panels E and F show staining of a section from a different MCC tumor. Panel E shows CK20 staining (green secondary). Panel F shows staining MCV VLP staining (red secondary).

MCC tumor sections were co-stained with MCV VLP-specific rabbit serum and antibody CM2B4, which is specific for MCV T antigen [10]. To generate positive controls, HeLa cells were transfected with expression constructs encoding either MCV VP1 or MCV T antigen. The transfected cells were paraffin-embedded and sectioned in a manner analogous to the preparation of the MCC tumor sections. As seen in Figure 5, T antigen and VP1 were readily detectable in the appropriate HeLa control cells. MCC tumor cells stained positive for MCV T antigen but negative for MCV VP1. Some MCC tumor sections were co-stained with antibody to cytokeratin-20 (CK20, a histological marker of MCC tumor cells) instead of CM2B4. While CK20 was readily visualized in MCC tumor cells, VP1 was again not detected in the tumor cells (Figure 5). 10/10 MCV T antigen-positive MCC tumors analyzed displayed an absence of VP1 staining. The results indicate that most MCC tumors produce little or no MCV VP1.

### Development of a candidate MCV vaccine

To investigate the functional immunogenicity of MCV VLPs, serum from a rabbit inoculated with purified MCV VP1/VP2 VLPs was tested using the reporter vector neutralization assay. Hyperimmune serum from the animal displayed a neutralizing titer of 1.9 million±0.4 million (Figure 1, top panel).

Five mice were also administered MCV VLPs. Two of the mice received an initial prime of VLPs without adjuvant, while three other mice received the VLP prime in complete Freund's adjuvant. All the mice received a booster dose of VLPs in incomplete Freund's adjuvant. Mice receiving the unadjuvanted prime displayed neutralizing EC_50_ titers of 0.9 and 3.2 million, while the three mice receiving the priming dose with adjuvant displayed titers of 1.1, 1.1 and 1.6 million. The results show that MCV VP1/VP2 VLPs can elicit potent MCV vector-neutralizing antibody responses in a vaccine setting.

## Discussion

The results show that, while a majority of older adults are exposed to MCV, the magnitude of serological responsiveness to the viral capsid proteins varies continuously across a 10,000-fold range. Compared to control subjects, all MCV^+^ MCC patients in the study displayed unusually high-titer humoral responses to MCV. In an initial EIA-based study establishing the prevalence of serological responsiveness to MCV in human subjects, we found that sera from MCV^+^ MCC patients contained MCV-specific antibodies at levels that appeared to saturate the EIA at the tested 1∶500 serum dilution [12]. EIA-saturating responses were less common among various groups of control subjects. In the current report we extend these observations, providing accurate scalar measurements of human seroresponsiveness to MCV.

The human polyomaviruses BKV and JCV are thought to establish latent infections that persist for decades [30]. For these virus types, reactivation from latency and active shedding of virions, which can occur under conditions of immunosuppresion, is positively correlated with serum antibody responses to the viral capsid proteins [27],[31],[32]. Thus, strong seroresponsiveness against MCV may record a history at some point of relatively uncontrolled MCV infection. Although it seems paradoxical that MCV infection would not be controlled by antibody responses expected to neutralize the infectivity of the virus, it is possible to imagine that MCV, like BKV and JCV, is able to establish a reservoir of latently infected cells. Such latent infections might be resistant to clearance by neutralizing antibodies and thus could serve as a durable source of immunogenic virions, even in the face of effective neutralizing antibody responses. Alternatively, a putative delayed immune response might have resulted in a high viral load that ultimately did induce high antibody levels. Since responses to BKV were similar in MCC patients and control subjects, it appears that MCC is associated with a specific failure to control MCV infection, as opposed to a more generalized failure to control all polyomavirus infections.

It is important to note that about a third of control subjects we studied displayed MCV responsiveness in the same range as MCV^+^ MCC patients (Figure 4). In light of the rarity of MCC (roughly 1500 cases per year in the United States, [33] reviewed in [34]), the results imply that most individuals who mount strong serological responses against MCV will not ultimately develop MCC. This is reminiscent of data indicating that exposure to ultraviolet light correlates with (but obviously does not guarantee) the development of MCC (reviewed in [4],[6]). Taken together, the results suggest a model in which uncontrolled MCV infection is one of multiple carcinogenic insults underlying the development of most cases of MCC.

Although MCV DNA has been detected in skin, bowel, lymph node, and respiratory tract samples [2], [35]–[37] the normal site or sites of productive MCV replication and the character of actively replicating MCV strains remains unclear. It is also unclear whether MCV infection may be a factor in other forms of disease in addition to MCC. MCV DNA sequences have recently been detected in a fraction of non-melanoma, non-MCC skin cancers, but a causal link between MCV and these forms of cancer has not yet been clearly established [38],[39]. While it is formally possible that neutralization of authentic MCV in the bona fide cellular target might differ with neutralization in 293TT cells, our results suggest that the current assay provides quantitative analysis of seroreactivity to a large subset of MCV neutralizing antibodies as reflected by the high rates of seropositivity detected in both MCC patients and in the general population. This assay can reveal potential links between the immunogenic infection with the virus and a disease state, such as MCC. Compared to VLP-based EIAs, the neutralization assay presented in this work demands less operator hands-on time and provides substantially more accurate results. Thus, the neutralization assay should become a preferred technique for investigating possible correlations between highly immunogenic MCV exposure and other disease states, including non-MCC cancers. It may be possible to increase the throughput of the assay by initially identifying high-titer subjects using a single serum dilution point. For example, a cutoff of 90% neutralization at the 1,600-fold serum dilution would have correctly identified all subjects with EC_50_ titer values greater than 20,000.

The apparent absence or very low level of VP1 protein expression we have observed in MCC tumors confirms previous suggestions that the virus does not actively replicate in MCC tumors. The finding is also consistent with the concept that the tumors are under immunological pressure favoring reduced expression of capsid proteins. This reduced expression could be due either to mutations in VP1, as appears to be the case for MCV350, or due to control of VP1 expression at transcriptional, RNA processing or translational levels. In any event, it appears to be unlikely that robust MCV capsid-specific antibody responses are directly primed by the MCC tumor, suggesting that strong seroresponsiveness to MCV indicates a prior history of active MCV infection of non-tumor or (pre-tumorous) tissues.

In human papillomavirus (HPV) infections, the virally induced cellular changes that lead to development of cancer occur in the absence of a productive viral infection and in the presence of existing neutralizing antibodies. A prophylactic HPV VLP-based vaccine that generates neutralizing antibodies seems to be sufficient to block the development of cancer by preventing the initial establishment of infection [40]. Development of MCC likewise seems to occur in the presence of effective humoral responses, but a prophylactic vaccine might nevertheless be effective for preventing the initial establishment or dissemination of MCV infection. In addition, more research might unveil MCV as a causative agent in more common public health threats, if so, a prophylactic vaccine might be beneficial. To begin to explore the idea that a VLP-based vaccine against MCV might be effective, we immunized animals with a candidate MCV vaccine composed of MCV VP1/VP2 VLPs. All the vaccinated animals displayed strong MCV vector-neutralizing antibody responses, with 50% neutralizing titers of roughly one million-fold serum dilution. This is comparable to the titers of animals administered HPV VLP-based vaccines [41], and higher than titers observed in animals receiving JCV VLPs, particularly when the JCV VLPs were administered without adjuvant [14]. Thus, it appears that MCV VLPs are relatively potent immunogens that could, in principle, be incorporated into existing VLP-based preventive vaccine regimens.

Cell culture and small animal models for MCV replication are not yet available and little is known about the infectious tropism of the virus beyond the clinical inference that it can enter Merkel cells or their precursors. To the extent that MCV reporter vector-mediated transduction may faithfully recapitulate the MCV infectious entry pathway, the vectors could be useful for exploring the entry tropism of the virus in vitro and in vivo. The vectors should also be useful for investigation of MCV virion assembly and structure, as well as for high-yield production of infectious virions containing MCV genomic DNA.

## Materials and Methods

### Ethics statement

This study was conducted according to the principles expressed in the Declaration of Helsinki. All samples and data for MCC patients were collected after written consent under study protocols approved by the institutional review boards of the University of Pittsburgh Cancer Institute and the University Clinic of Würzberg. For control individuals consent was not obtained, instead samples were de-identified and analyzed anonymously.

All animal experiments were performed at Lampire (Pipersville, PA) commercial facilities. Protocols at this facility are reviewed and approved for use by the Lampire Institutional Animal Care and Use Committee (IACUC) as mandated for a USDA regulated research institution.

### Virus production

MCV reporter vector stocks were produced by transfecting human embryonic kidney cells engineered to stably express the cDNA of SV40 T antigen (293TT) [15]. The cells were transfected using Lipofectamine2000 (Invitrogen) according to previously-reported methods [42]. In initial studies, plasmids pwM and ph2m [12] expressing, respectively, codon-modified versions of the VP1 and VP2 genes of MCV strain 339, were co-transfected with a GFP reporter plasmid, pEGFP-N1 (Clontech). Neutralization assay stocks employed phGluc, which encodes a *Gaussia* luciferase reporter gene (NEB), as a reporter plasmid. Forty-eight hours after transfection, the cells were harvested and lysed at high density (10^8^ cells per ml) in Dulbecco's phosphate buffered saline (DPBS, Invitrogen) supplemented with 9.5 mM MgCl_2_, 0.4% Triton X-100 (Pierce), 0.1% RNase A/T1 cocktail (Ambion) and antibiotic-antimycotic (Invitrogen). The cell lysate was incubated at 37°C overnight with the goal of promoting capsid maturation [43]. Lysates containing mature capsids were clarified by centrifugation for 10 min at 5000×g. The clarified supernatant was loaded onto a 27–33–39% iodixanol (Optiprep, Sigma) step gradient prepared in DPBS with a total of 0.8 M NaCl. The gradients were ultracentrifuged 3.5 hours in an SW55 rotor at 50,000 rpm (234,000×g). Gradient fractions were screened for the presence of encapsidated DNA using Quant-iT Picogreen dsDNA Reagent (Invitrogen). VP1 protein concentration was determined by comparing vector stock to bovine serum albumin standard (BioRad) in SYPRO Ruby-stained Nupage gels (Invitrogen). Vector stock yields were typically several µg of purified VP1 per 225 cm^2^ flask of transfected cells.

Vector stocks based on murine polyomavirus (MPyV) or BKV were produced using a similar scheme. For MPyV cells were co-transfected with plasmids pwP and ph2p [12] (carrying codon-modified MPyV VP1 and VP2, respectively) together with phGluc. An additional plasmid, ph3p, encoding the MPyV minor capsid protein VP3, was also included in the co-transfection mixture. For BKV vector stocks, plasmid pCAG-BKV (a generous gift from Dr. Akira Nakanishi (NCGG, Japan) [28]) encoding the capsid protein genes was co-transfected with phGluc.

In some virion production systems, capsids containing linear fragments of cellular DNA can substantially outnumber capsids containing the viral genome or desired reporter plasmid [43],[44]. In the vector harvest procedure detailed above, unwanted capsids associated with large segments of cellular DNA (as opposed to reporter plasmid DNA) tend to sediment away during the 5000×g clarification step and tend to be retained toward the top of the Optiprep gradient ([42] and unpublished results). For production of VLPs, recovery of capsids containing cellular DNA is desirable and was achieved by adding Benzonase (Sigma) and Plasmid Safe (Epicentre) nucleases to the lysis buffer (0.1% each) and adjusting the lysate to 0.8 M NaCl immediately prior to clarification. These modifications to the harvest protocol increased VLP yield to roughly 1 mg of VP1 per transfected 225 cm^2^ flask.

Maps of plasmids used in this work and detailed virus production protocols are available from our laboratory website <http://home.ccr.cancer.gov/LCO/>.

### Neutralization assay

Neutralization assays were performed using a 96-well plate format. Sera and virus stocks were diluted in cell culture medium (DMEM without phenol red and supplemented with 25 mM HEPES, 10% heat-inactivated fetal bovine serum, 1% MEM non-essential amino acids, 1% Glutamax and 1% antibiotic-antimycotic, all from Invitrogen). Test sera were subjected to a series of ten four-fold dilutions (range 1∶100 to 1∶2.6×10^7^). 24 µl of the diluted serum sample were added to 96 µl of diluted reporter vector stock. The virus/diluted serum mixture was gently agitated then placed on ice for 1 hour. 293TT cells were seeded in 100 µl of culture medium at a density of 3×10^4^ cells/well in 96-well flat bottom plates for 3–5 hours prior to addition of 100 µl of the virus/serum mixture. Each plate also contained eight wells of cells receiving vector stock without test serum (no serum control) and 2 wells with cells that received only culture medium (no virus control). To minimize plate edge effects, the outer wells of the plate were not used for the assay and were instead filled with culture medium. Three days after virus inoculation, the plates were thoroughly agitated and 25 µl samples of conditioned culture supernatant were transferred to a white 96-well luminometry plate (Perkin Elmer). A BMG Labtech Polarstar Optima luminometer was used to inject 50 µl of *Gaussia* Luciferase Assay Kit substrate (NEB), and light emission (in relative light units, RLUs) was measured according to manufacturer instructions. Typical assay conditions resulted in a “no serum” signal of roughly 500,000 RLUs with a “no virus” noise of <500 RLUs.

To calculate effective concentration 50% (EC_50_) values, Prism software (GraphPad) was used to fit a variable slope sigmoidal dose-response curve to RLU values for each serum dilution series. Curves were constrained to average no serum and no virus control values. Each serum sample was tested in at least two independent neutralization assay runs. A small subset of sera whose repeat EC_50_ values differed by more than three-fold were re-tested until their EC_50_ values stabilized. For all sera, the results of the final round of testing are shown. Although the sera used in this work were not heat-inactivated prior to testing, analysis of a subset of human sera showed that the assay is compatible with a 30 minute 56°C heat-inactivation of test sera (data not shown).

BKV neutralization assays were performed using 293TT cells with a dose of less than 50 pg of VP1 per well. The MPyV neutralization assay was performed using 293TT cells in a similar fashion except that sera were tested at a single dilution (1∶500) against an MPyV-Gluc vector. The MPyV-Gluc vector transduced 293TT cells and murine NIH-3T3 cells much less efficiently than the MCV-Gluc vector and it was therefore necessary to use a dose of 2 ng of MPyV VP1 per well. The MPyV neutralization assay was carried out in the presence of 100 nM trichostatin A (EMD Biosciences), a histone deacetylase inhibitor that has previously been shown to enhance MPyV vector-mediated transduction [45].

### EIAs

EIAs were performed using Immulon HB2 plates (Thermo) coated overnight with VLPs at 100 ng/well in PBS. The wells were blocked with PBS+0.5% nonfat dry milk (blotto). Serum samples were diluted in blotto and incubated in EIA wells at room temperature with orbital shaking for 45 minutes. The plates were then washed with PBS and bound antibody was detected using horseradish peroxidase-conjugated donkey anti-human IgG (Jackson) diluted 1∶7500 in blotto. ABTS substrate (Roche) development was monitored by absorbance at 405 nm with a reference read at 490 nm.

### Immunofluorescence staining

Merkel Cell carcinoma tissue sections were cut from formalin-fixed paraffin embedded biopsies collected under a University of Pittsburgh IRB approved protocol. Staining was performed as described by Robertson et al. [46] with some modifications. Briefly, slides with the formalin fixed paraffin embedded tissues were baked for 1 hour at 60°C. Deparaffinization was performed by rinsing twice in xylenes for 5 min, once for 30 seconds in each of the following solutions: 100% Ethanol, 90% ethanol, 70% Ethanol, and twice in deionized water for 30 seconds. Slides were then placed in a jar containing 1× Target Retrieval Solution (Dako # S6199) in a 95 degree water bath for 30 minutes. The jar was then incubated at room temperature for 20 minutes and the slides rinsed 3 times for 1 min in water. Sections were blocked for 10 min at 37 degrees in Protein Block solution (Dako #x0909), incubated in primary antibody for 2 hours at 37 degrees, rinsed 3 times in PBS, and incubated in Alexa-488 or 594 conjugated secondary antibodies at a 1∶1000 dilution (Invitrogen) followed by 3 rinses in PBS. Prolong Gold Antifade with Dapi (Invitrogen) was used as the mounting medium and slides were visualized by Confocal microscopy using a Zeiss NLO 510 instrument. The primary antibodies were Mouse anti-cytokeratin (Dako) used at 1∶50, Purified anti-MCV T antigen monoclonal CM2B4 [10] at 1∶300, and rabbit anti-MCV (VP1/VP2) generated as described in “Candidate MCV Vaccine” section used at 1∶2000. Images of Hela controls (T Antigen and VP1/2 transfections) and MCC samples had identical gain and pinhole settings, however the gain was lowered by 30% on Hela control cells transfected with T antigen to remain in the linear range of pixel saturation.

### Sera

A pool of human sera from male U.S. AB plasma donors was purchased from Sigma (cat# H4522). IgG was purified out of the pooled sera using a Pierce NAb Protein G Kit, according to manufacturer's instructions. To generate a neutralization curve, the purified IgG (1.1 mg/ml) was standardized to the IgG content of the original serum (8.1 mg/ml). Serum was stripped of immunoglobulins by passage over a mixture of protein L and protein A/G resins (Pierce).

De-identified blood donor sera were obtained from the Columbia University and New York City Blood Banks. Individual serum samples from paid donors visiting U.S. plasma donation centers were purchased from Equitech-Bio and Innovative Research. The paid donors were 69% male, 42% Caucasian, 56% African American, and had a mean age of 56 years (range 47–75). All sera were tested for antibodies against HIV, HCV, HBV and syphilis and were found to be negative. 21 MCV positive cases (age 14–95 years) were obtained from persons with histologically-confirmed MCC [12]. MCV status was determined by qPCR as previously described [2].

### Candidate MCV vaccine

To generate MCV-specific serum, a rabbit was immunized with two 300 µg doses of MCV VP1/VP2 VLPs, according to a standard immunization schedule offered by Lampire, Inc. The first dose was prepared in complete Freund's adjuvant. A booster dose was administered 3 weeks later in incomplete Freund's adjuvant. Immune serum was collected 10 days after the boost. Mice were immunized twice with 80 µg of MCV VP1/VP2 VLPs. For three mice, the first dose was prepared in complete Freund's adjuvant. Another two mice were primed with VLPs in PBS without adjuvant. For all mice, the boost (4 weeks post-prime) was administered in incomplete Freund's adjuvant. Sera were collected for testing 10 days after boosting. Rabbit serum specific for MPyV VP1 was a generous gift from the lab of Dr. Thomas L. Benjamin (Harvard) [18].

## Supporting Information

Figure S1 MCV350 VP1 is defective. 293TT cells transfected with expression plasmids encoding the VP1 proteins of MPyV, MCV339 or MCV350 were lysed with triton X-100, then incubated at 37°C for 20 minutes or overnight. The lysates were subjected to Western blotting with a rabbit serum specific for MCV VLPs. Western blotting for GFP, which is co-expressed by the VP1 expression plasmids, is shown in the bottom panel.
(0.22 MB PDF)

Figure S2 EIA versus neutralizing EC_50_ values for individual sera. Serum samples were serially diluted and tested in MCV VLP EIA or reporter vector neutralization assay. Calculated EC_50_ values for each serum sample are shown.
(0.20 MB PDF)

Figure S3 Age versus neutralizing EC_50_. The neutralizing EC_50_ values (y axis) for individual serum samples from control subjects or MCV+ MCC patients are plotted against donor age (in years, x axis). Values for MCC patients whose tumors tested negative for MCV DNA are also shown.
(0.07 MB PDF)
