# Supplementary figures and images for: Quantitation of Human Seroresponsiveness to Merkel Cell Polyomavirus
Source: PLoS Pathog. 2009 Sep 11;5(9):e1000578. doi: 10.1371/journal.ppat.1000578 (PMC2734180; doi:10.1371/journal.ppat.1000578)

Supplemental Figure 1

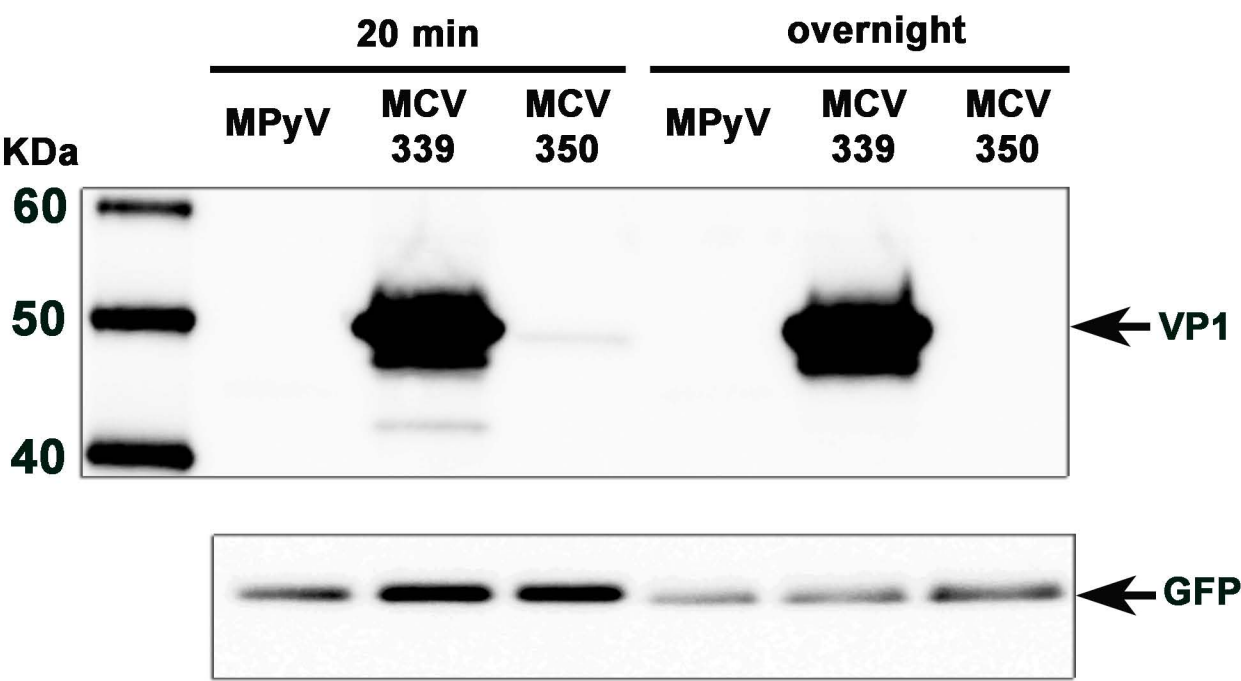

Supplement: Figure S1 — MCV350 VP1 is defective. 293TT cells transfected with expression plasmids encoding the VP1 proteins of MPyV, MCV339 or MCV350 were lysed with triton X-100, then incubated at 37°C for 20 minutes or overnight. The lysates were subjected to Western blotting with a rabbit serum specific for MCV VLPs. Western blotting for GFP, which is co-expressed by the VP1 expression plasmids, is shown in the bottom panel. (0.22 MB PDF) [file ppat.1000578.s001.pdf]

Supplemental Figure 2

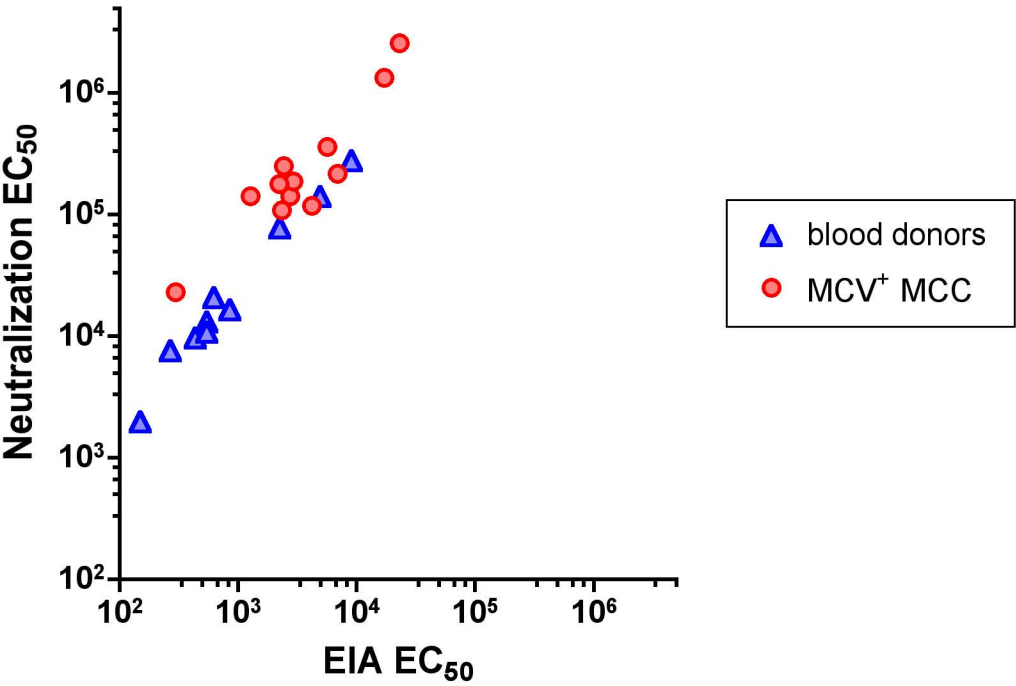

Supplement: Figure S2 — EIA versus neutralizing EC50 values for individual sera. Serum samples were serially diluted and tested in MCV VLP EIA or reporter vector neutralization assay. Calculated EC50 values for each serum sample are shown. (0.20 MB PDF) [file ppat.1000578.s002.pdf]

Supplemental Figure 3

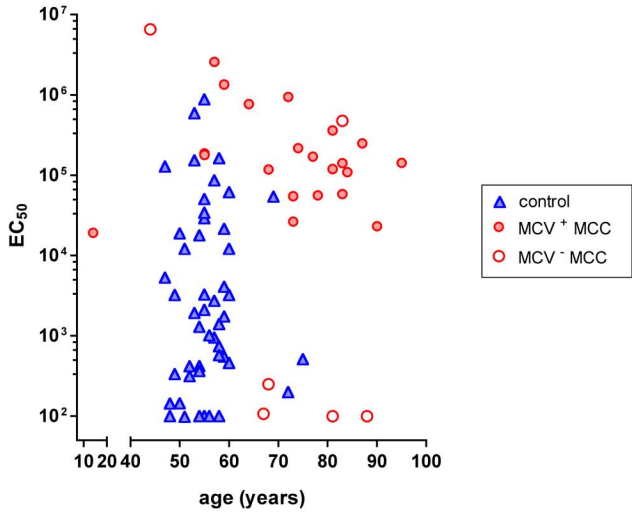

Supplement: Figure S3 — Age versus neutralizing EC50. The neutralizing EC50 values (y axis) for individual serum samples from control subjects or MCV+ MCC patients are plotted against donor age (in years, x axis). Values for MCC patients whose tumors tested negative for MCV DNA are also shown. (0.07 MB PDF) [file ppat.1000578.s003.pdf]
